# Supplementary material for: The genetics and epidemiology of N- and O-immunoglobulin A glycomics
Source: Genome Med. 2024 Aug 9;16:96. doi: 10.1186/s13073-024-01369-6 (PMC11312925; doi:10.1186/s13073-024-01369-6)
Supplement: Supplementary file 2 — Additional file 2. Supplementary Figures. [file 13073_2024_1369_MOESM2_ESM.docx]

**Supplementary Figures for:**

***The genetics and epidemiology of N- and O- Immunoglobulin A glycomics***

Alessia Visconti^1,2,†^, Niccolò Rossi^1, †^, Albert Bondt^3^, Agnes Hipgrave Ederveen^3^, Gaurav Thareja^4^, Carolien A. M. Koeleman^3^, Nisha Stephan^4^, Anna Halama^4^, Hannah J. Lomax-Browne^5^, Matthew C. Pickering^5^, Xu-jie Zhou^6,7,8,9^, Manfred Wuhrer^3,#^, Karsten Suhre^4,#^, Mario Falchi^1,#^

^1^Department of Twin Research and Genetic Epidemiology, King’s College London, London, UK

^2^Center of Biostatistics, Epidemiology and Public Health, Department of Clinical and Biological Sciences, University of Turin, Turin, Italy

^3^Center for Proteomics and Metabolomics, Leiden University Medical Center, Leiden, The Netherlands

^4^Department of Biophysics and Physiology, Weill Cornell Medicine–Qatar, Doha, Qatar

^5^Centre for Inflammatory Disease, Department of Immunology and Inflammation, Imperial College London, London, UK

^6^Renal Division, Peking University First Hospital, Beijing, China.

^7^Peking University Institute of Nephrology, Beijing, China.

^8^Key Laboratory of Renal Disease, Ministry of Health of China, Beijing, China.

^9^Key Laboratory of Chronic Kidney Disease Prevention and Treatment, Peking University, Ministry of Education, Beijing, China.

^†^These authors contributed equally

^#^These authors share senior authorship


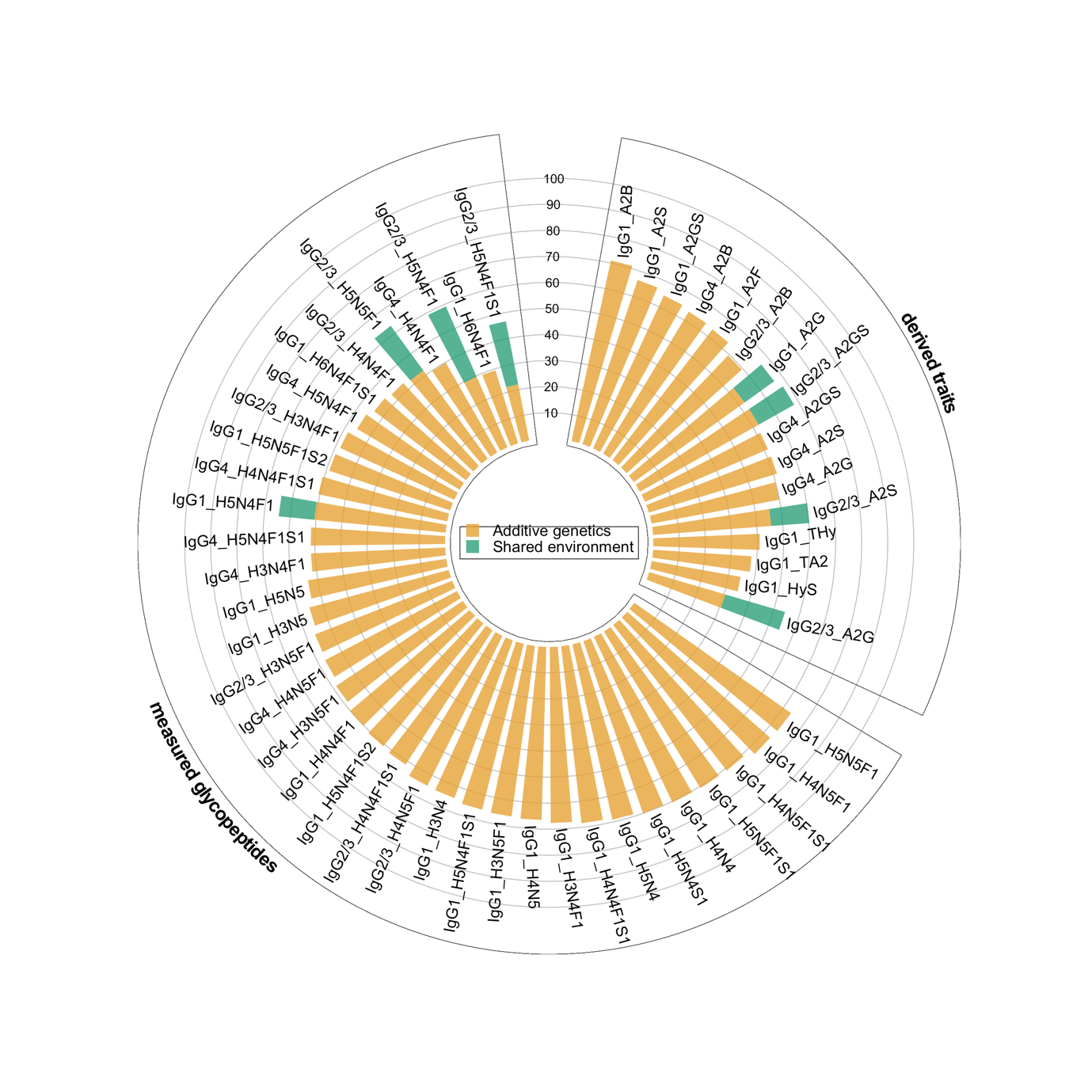


**Fig S1**. **Heritability of IgG measured glycopeptides and derived traits.** We used the ACE model to partition the variance for each glycan trait into additive genetic (orange), and shared (green) and unique (white) environmental components.


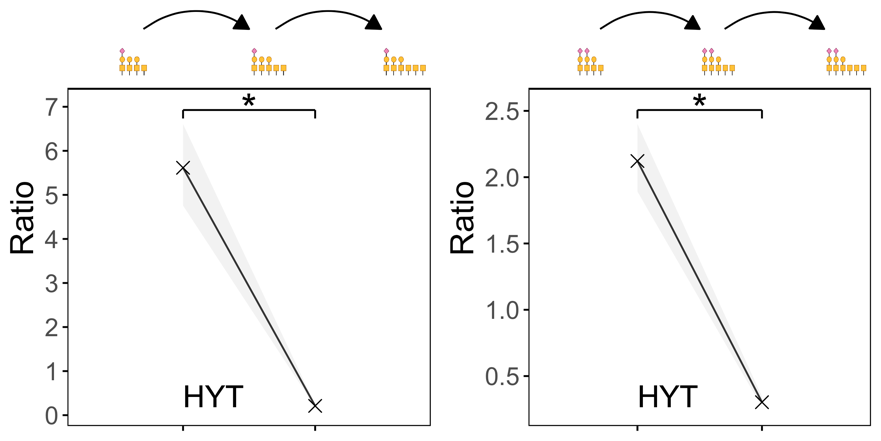


**Fig S2. Progressive decrease in GalNAc transfer efficiency of two different growing *O*-glycan structures on IgA1.** Crosses represent the median value of the ratio of the glycan structures depicted on top of the panel at the corresponding position of the x-axis. These glycan structures differ for a single GalNAc residue, and thus reflect sequential transfer of GalNAc residues in the *O*-glycosylation pathway. The grey area shows the interquartile range of each ratio. Significative differences, evaluated by means of the Wilcoxon test, are indicated with an asterisk (*P*<2.2×10^-16^)


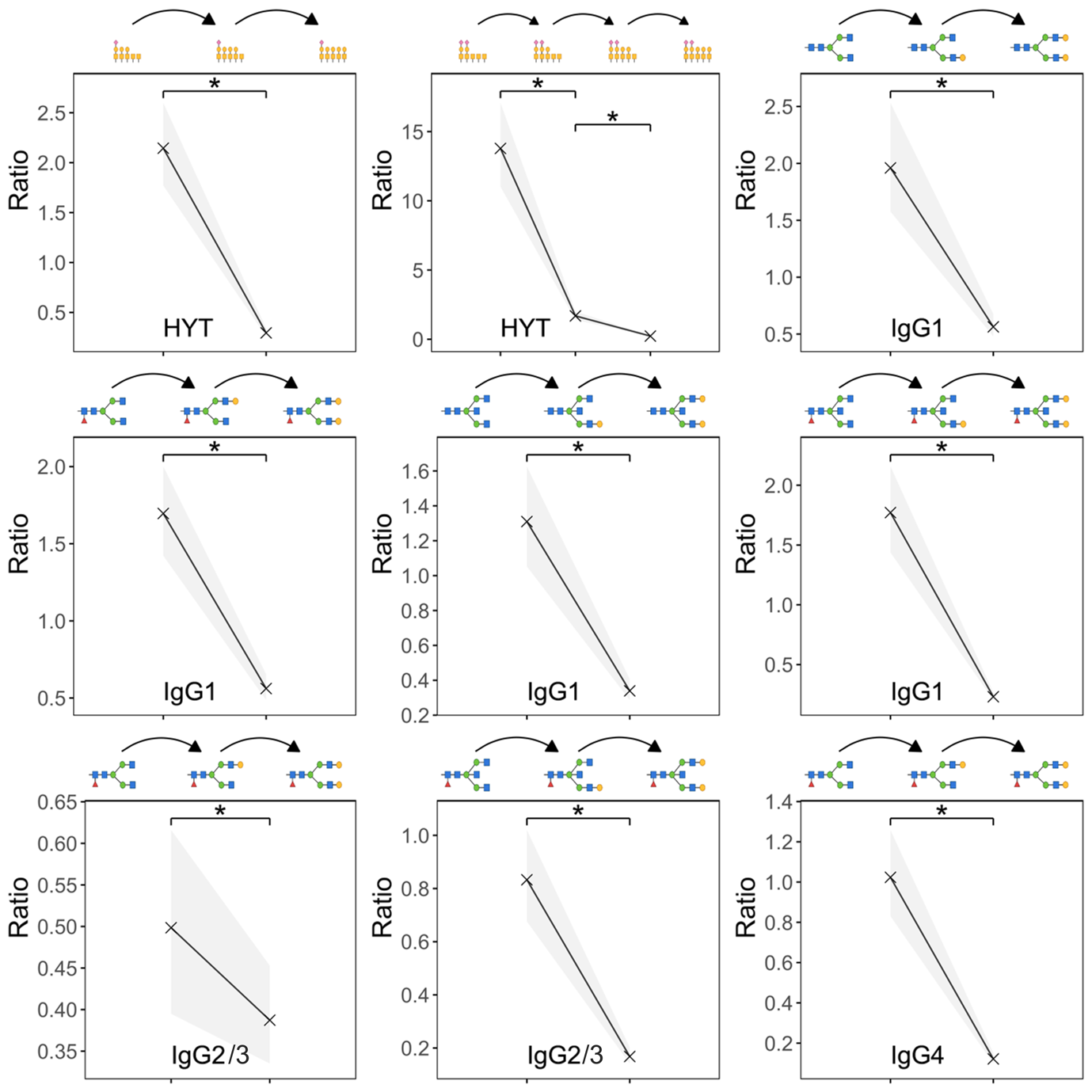


**Fig S3. Progressive decrease in galactosylation efficiency of growing *O*- and *N*-glycan structures on IgA and IgG, respectively.** Crosses represent the median value of the ratio of the glycan structures depicted on top of the panel at the corresponding position of the x-axis. These glycan structures differ for a single galactose residue, and thus reflect sequential galactosylation reactions in the glycosylation pathway. The grey area shows the interquartile range of each ratio. Significative differences, evaluated by means of the Wilcoxon test, are indicated with an asterisk (*P*<2.2×10^-16^).


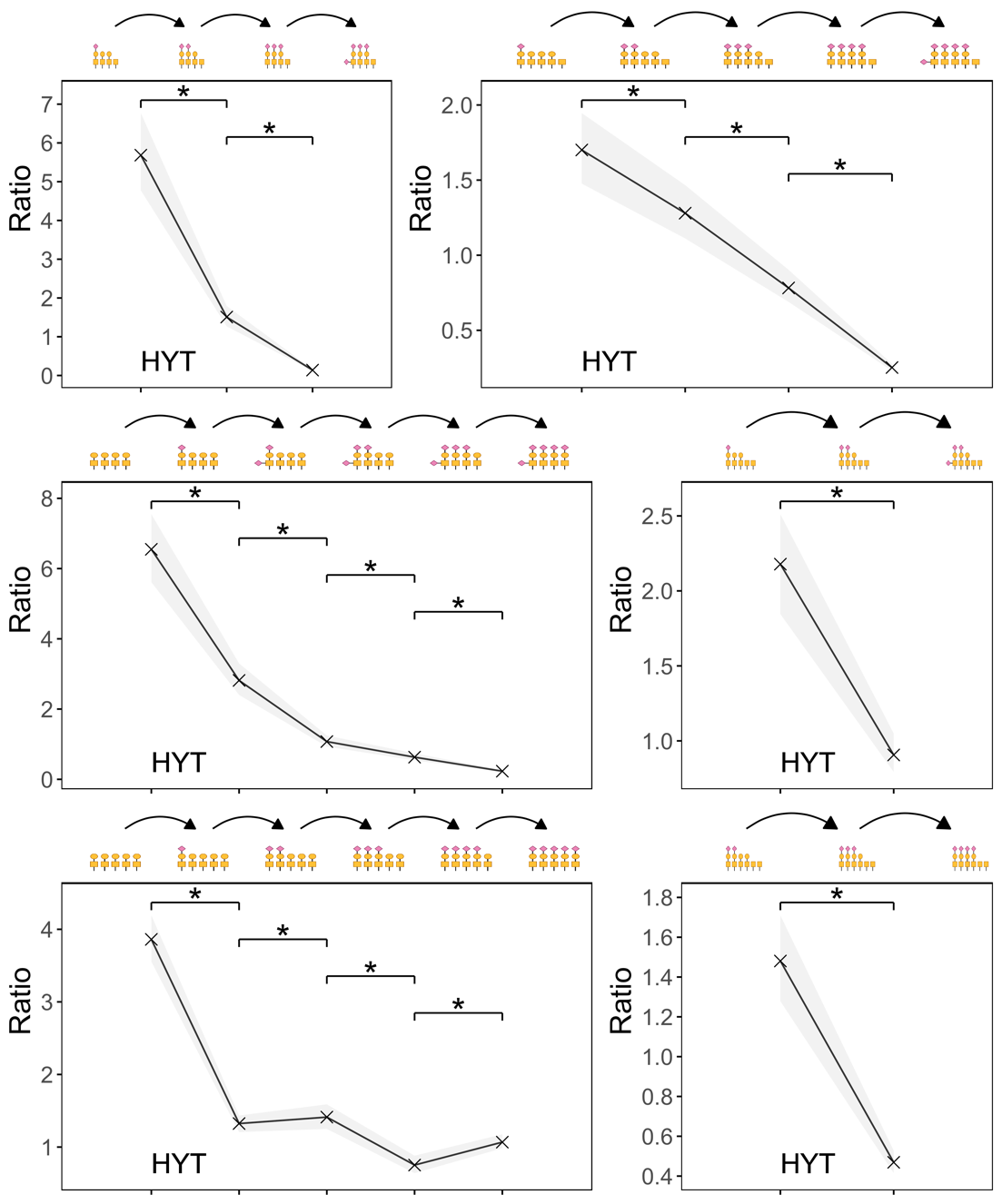


**Fig S4. Progressive decrease in sialylation efficiency of growing *O*-glycan structures on IgA_1_.** Crosses represent the median value of the ratio of the glycan structures depicted on top of the panel at the corresponding position of the x-axis. These glycan structures differ for a single sialic acid residue, and thus reflect sequential sialylation reactions in the *O*-glycosylation pathway. The grey area shows the interquartile range of each ratio. Significative differences, evaluated by means of the Wilcoxon test, are indicated with an asterisk (*P*<2.2×10^-16^).


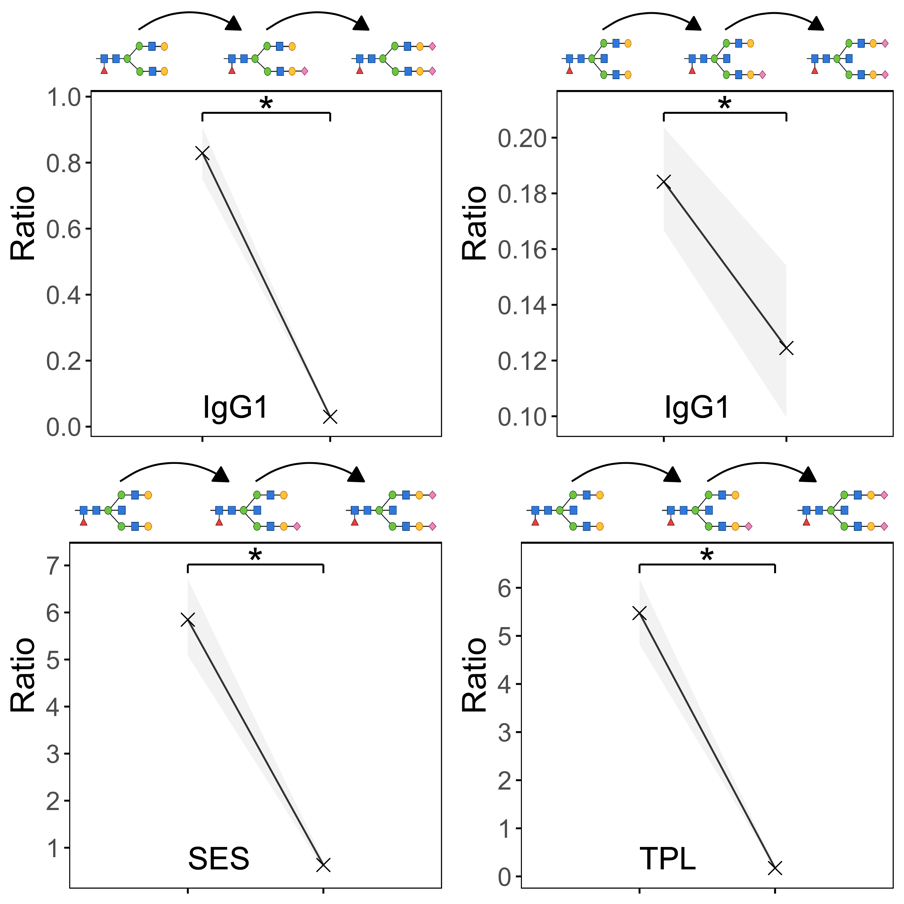


**Fig S5. Progressive decrease in sialylation efficiency of growing *N*-glycan structures on IgA and IgG.** Crosses represent the median value of the ratio of the glycan structures depicted on top of the panel at the corresponding position of the x-axis. These glycan structures differ for a single sialic acid residue, and thus reflect sequential sialylation reactions in the *N*-glycosylation pathway. The grey area shows the interquartile range of each ratio. Significative differences, evaluated by means of the Wilcoxon test, are indicated with an asterisk (*P*<2.2×10^-16^).


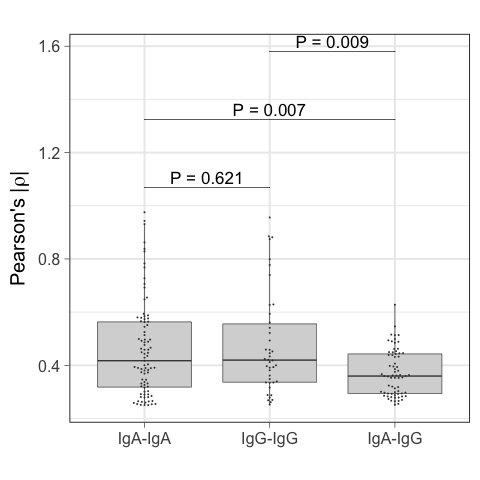


**Fig S6. Distribution of significant intra- and inter-correlation coefficients.** The plot shows the distribution of the absolute pairwise correlation coefficients which were significant, *i.e.*, passed a Bonferroni-derived threshold and had *|𝜌|>0.25*.  P values were calculated *via* Wilcoxon test.
